# Connection to... Addressing Digital Inequities in Supporting the Well-Being of Young Indigenous Australians in the Wake of COVID-19

**DOI:** 10.3390/ijerph18042141

**Published:** 2021-02-22

**Authors:** Roz Walker, Kim Usher, Debra Jackson, Corinne Reid, Katrina Hopkins, Carrington Shepherd, Reakeeta Smallwood, Rhonda Marriott

**Affiliations:** 1 Ngangk Yira Research Centre for Aboriginal Health and Social Equity, Murdoch University, Murdoch, WA 6150, Australia; kusher@une.edu.au (K.U.); Debra.Jackson@uts.edu.au (D.J.); kdhopkins@bigpond.com (K.H.); carrington.shepherd@curtin.edu.au (C.S.); R.Marriott@murdoch.edu.au (R.M.); 2 School of Indigenous Studies, The University of Western Australia, Crawley, WA 6009, Australia; 3 School of Health, University of New England, Armidale, NSW 2351, Australia; 4 Susan Wakil School of Nursing, University of Sydney, Sydney, NSW 2006, Australia; Reakeeta.L.Smallwood@student.uts.edu.au; 5 Chancellory, Victoria University, Footscray, VIC 3011, Australia; corinne.reid@vu.edu.au; 6 Curtin Medical School, Curtin University, Bentley, WA 6102, Australia; 7 School of Nursing and Midwifery, Faculty of Health, University of Technology, Ultimo, NSW 2007, Australia

**Keywords:** Indigenous, social and emotional well-being, mental health, digital technologies, young people, equitable access, culture

## Abstract

(1) *Background:* This article examines whether connection to digital technologies helps connect young Indigenous people in Australia to culture, community and country to support good mental health and well-being and protect against indirect and potentially long-term effects of COVID-19. (2) *Method:* We reviewed literature published between February and November 2020 and policy responses related to digital strategies. We searched PubMed, Google Scholar, government policy websites and key Indigenous literature sources, identifying 3460 articles. Of these, 30 articles and 26 policy documents were included and analysed to identify existing and expected mental health outcomes among Indigenous young people associated with COVID-19 and more broadly. (3) *Results:* There are inequities in affordable access to digital technologies. Only 63% of Indigenous people have access to internet at home. Digital technologies and social media contribute to strong cultural identity, enhance connections to community and country and improve mental health and social and emotional well-being outcomes. (4) *Discussion:* Access to digital technologies can facilitate healing and cultural continuity, self-determination and empowerment for young people to thrive, not just survive, in the future. (5) *Conclusion:* More targeted policies and funding is urgently needed to promote digital technologies to enhance Indigenous young people’s access to mental health and well-being services, maintain cultural connections and evaluate the effectiveness of these initiatives using Indigenous well-being indicators.

## 1. Introduction

This paper aims to examine whether utilising digital technologies helps connect young Indigenous people in Australia to culture, community and country to support good mental health and well-being as well as protect against indirect and potentially long-term effects of COVID-19. Since the first case of COVID-19 was reported in Australia in February 2020 there have been over 28,800 COVID-19 cases, 66 active cases and 909 deaths recorded in Australia, one of the least impacted countries in the world [1]. The Northern Territory, Australian Capital Territory and Tasmania have remained almost COVID-19 free while states such as Victoria, New South Wales and Queensland have experienced further waves of the pandemic [1].

While Australia appears to have avoided the worst of the COVID-19 pandemic, there remains the looming threat of a second (and in some states a third) wave of infections and associated deaths as well as associated disabilities referred to as ‘long COVID’ [2,3]. In addition, in the wake of the prolonged first wave, there are fears that unemployment levels, financial uncertainty and mental health issues including anxiety, depression and self-harm will increase in communities around Australia, along with indirect effects on physical health [4,5]. The emerging global evidence highlights that the burden of COVID-19 is not equally shared among population groups within countries. Marginalised and ethnic minority communities have been hardest hit. Communities living in poverty, such as Indigenous communities, have high rates of underlying health conditions, poor access to healthcare and life circumstances that make self-isolation difficult [6,7]. In these communities, COVID-19 is not simply a pandemic—it is a syndemic. That is, there are interacting biological (COVID-19, comorbid health conditions) and social factors (poverty, poor housing, etc.) that create serious and enduring multiplicative, compound effects [5,8].

This is especially relevant in the Australian context, where a range of distal social determinants that form part of a complex colonial legacy have contributed significantly to these adverse circumstances and issues for Indigenous peoples. These determinants include unequal access to health services, education and employment, and persistent experiences of institutional and interpersonal racism [5,9,10], as well as a failure to recognise Indigenous sovereignty within the constitution. These political and social processes of colonisation have resulted in forms of historical trauma that have been transmitted through individuals, families and communities over generations. For many Indigenous people, including young people, the ongoing legacies of colonisation contribute to forms of complex and compounding trauma [11,12,13].

It is estimated that prior to colonisation there were over 500 different Indigenous nations with distinctive cultures, beliefs and languages across Australia [14]. In 2016, there were an estimated 798,365 Aboriginal and Torres Strait Islander people (herein respectfully referred to as Indigenous peoples) in Australia (91% Aboriginal, 5% Torres Strait Islander and 4% both), comprising 3.3% of the total Australian population [15]. The Indigenous population is much younger, with a medium age of 20 years compared with 35 years for other Australians [16]. Indigenous young people under 25 years comprise 44% percent of the Indigenous population compared to 24.4% for non-Indigenous people [15]. The majority of Indigenous and non-Indigenous people live in urban and regional areas (98% and 81% respectively); however, there are distinct variations across Australian states and territories. For instance, approximately 77% of Indigenous people in the Northern Territory live in remote and very remote areas with limited services and internet facilities, compared with just 4% in New South Wales [17]. In general, Aboriginal people who live in remote areas continue to maintain their cultural traditions and languages [16]. Census data from 2016 reveal around 55% of Aboriginal people living in remote areas speak an Indigenous language compared to 8% of those living in non-remote areas. Overall, the census found around 150 Indigenous languages were spoken in Australian homes [17]. Importantly, language plays a crucial role in Indigenous sovereignty along with spiritual ways, cultural, social and political systems/structures, lore/laws and innate relationships with lands, waters and the cosmos. Enduring Indigenous knowledges affirm that interrelationships exist as long as Indigenous peoples continue to identify with specific nations/communities. Few studies to date have focused specifically on the impact of COVID-19 on Indigenous peoples, who are among the most vulnerable of population groups in almost every measurable aspect of well-being and, therefore, potentially more susceptible to the ongoing impacts of the current pandemic. A survey of acute mental health responses during the COVID-19 pandemic suggests Indigenous peoples experienced higher levels of psychological distress, anxiety and stress than other Australians [18]. These responses make Indigenous peoples vulnerable to poorer mental health influencing their behavioural responses to COVID-19. Importantly, recent studies have shown that digital technologies and social media platforms are both acceptable and effective in supporting mental health and well-being to help mitigate likely impacts for young Indigenous people and, therefore, are a crucial component of the COVID-19 response [19].

Many Indigenous communities, like many non-Indigenous communities in Australia, are experiencing a sense of disconnection. There is an urgent need for strategies to support vulnerable communities experiencing uncertainty associated with the pandemic as well as with the social and lifestyle restrictions imposed in order to halt the spread of the infection. Young Indigenous people face elevated population-level risks of psychological distress, suicide and health-harming behaviours [4]. There has been a reported increase in the number of people accessing crisis lines and mental health services due to COVID-19 [20,21]. Suicide rates and clusters among Indigenous young people are especially concerning and require additional support through e-health apps and age-appropriate social media that have previously proven effective in reducing suicide and self-harm [22]. Importantly, Indigenous young people demonstrate strong adaptability in their engagement with new technologies to support culturally responsive mental health interventions [23]. Several studies have shown that digital technologies and platforms such as Facebook© can promote connection to family and community, provide cultural continuity and help young people to stay involved with friends [24,25,26]. Facilitating this level of support, connection and community via social media can help maintain young people’s mental health and well-being [25]. Indigenous-led digital responses that support connectivity and promote resilience include events such as a virtual concert streamed from North East Arnhem Land, which connected people through song and dance [27] as well as through the development of contextually relevant e-medical services [28]. Such examples highlight the positive potential for innovative, culturally responsive and effective digital strategies to overcome the inequities in access for young people, their families and communities.

Locally, the strength of these communities and importance of connection to culture and country has also been displayed in powerful “place-based” COVID-19 responses, highlighting Indigenous self-determination and resilience. Several studies demonstrate how digital technologies and social media contribute to strong cultural identity; enhance connections to community and country and promote health and social and emotional well-being (SEWB) outcomes [24,25,29]. Importantly, these studies show how access to digital technologies has the potential to facilitate healing, cultural continuity, self-determination and empowerment as well as address existing inequities in access to health services. Similarly, it has been found that social media also enhanced strong cultural identity and community and family connections linked to improved educational and health outcomes [16]. Strategies to support social connection and connection to culture, community and country, especially during times of extended trauma, anxiety, grief and loss, are urgently needed to promote SEWB and ensure resilience is maintained.

While this review examines the potential positive impacts of digital technology and social media on enhancing cultural connections and addressing social inequities, several studies also highlight the negative impacts. For instance, a study by Indigenous researcher, Bronwyn Carlson raises concerns about the likely trauma experienced by Indigenous young people due to the frequent exposure to untimely deaths, suicide, deaths in custody, violence and funerals being shared on social media [30]. Carlson reports Indigenous people can also experience an unrelenting sadness and sorrow through increased exposure to new mourning practices by way of sorry pages on Facebook©.

A systematic review examining the positive and negative impacts of Indigenous young people’s use of digital technologies and social media found that “cyber bullying, cyber racism and the exchange of sexually explicit content between minors are common with limited approaches to dealing with this at the community level” (p. 1, [16)]. According to a recent review, cyberbullying among Indigenous young people is widespread, experienced at different rates and for different reasons than in mainstream populations [31]. These authors argue there are a range of cultural, social and political factors that influence the rates of, kinds of, and responses to cyberbullying among Indigenous peoples. For example, some evidence indicates cyberbullying is increasing in remote communities where young people are ignoring traditional communication practices [32]. In addition, cyberbullying has been linked to suicide, suicide ideation, depression, anxiety and other mental health issues in young people [33]. Carlson and Frazer argue the need for greater understanding of the causes of cyberbullying as something that “occurs within and across families, clans and communities, rather than just between individuals”, in order to increase “understandings of its causes and impacts and how these might be mitigated” (p. 15, [31]), as well as strategies to effectively address it.

This literature review examines the benefits of digital technologies to support Indigenous SEWB. The results will be especially relevant in the context of the stress, risks and long-term impacts of the COVID-19 pandemic.

## 2. Materials and Methods

We are a group of Indigenous and non-Indigenous qualitative and quantitative researchers with a range of disciplinary and experiential expertise in Indigenous health, mental health and well-being. We conducted a comprehensive review of recent literature reviews, policy responses and relevant studies to examine both the existing and expected mental health outcomes among Indigenous young people as well as their access to, and utilisation of, digital technologies in addressing their mental health and SEWB in the context of COVID-19. We applied a narrative review to explore a new topic of interest not previously addressed [34].

### 2.1. Search Terms

We searched the HealthInfoNet; Lowitja Lit.Search access to the PubMed database; and Google Scholar. We used key terms for population groups (Indigenous OR Aboriginal, OR First Nation AND Young People), digital technology (e-health OR internet OR Social Media AND access) and SEWB (social and emotional well-being OR mental health), AND COVID-19 as an additional parameter of interest. We also scanned relevant media, government, academic and Indigenous-specific websites, Australian Policy Online and YouTube adding the additional search terms Indigenous, barriers AND communication to the previous terms.

Given the issues around mental health and given that inequitable access to services for Indigenous communities has escalated and become more obvious since COVID-19, we initially focused on peer-reviewed articles, grey literature including government and scientific research committee reports, working papers and policy documents published in 2020 between February and November and then searched relevant policy documents as well as additional publications referred to in the literature citations.

### 2.2. Inclusion Criteria

Inclusion criteria included articles referring to the use of digital technologies by Indigenous young people in the Australian context. The Lowitja PubMed search produced 580 relevant articles, and the Google Scholar search for articles during 2020 produced 2880 results. From these, 50 relevant articles were initially selected. We screened the titles and abstracts, and following removal of duplicates, reviewed 30 complete articles that discussed Indigenous young people’s use of the internet and/or social media for either social connection or for access to mental health services or tools for further assessment. A further extended search of relevant media, government, academic and Indigenous-specific websites, Australian Policy Online, YouTube and a scan of references resulted in 26 government policy reports, Indigenous committee reports and statistics that discussed strategies to address digital inequities and/or mental health impacts due to COVID and 32 relevant peer-reviewed articles examining the benefits and risks of social media for Indigenous young people and its role in connecting young people to culture. We grouped the issues into overall themes, all authors agreed on the main themes identified in this paper.

## 3. Results

A total of, 88 full texts were analysed [34]. The results of the review covered a range of themes, which have been grouped under four headings. These include the following: (1) inequities in access impacting existing and expected mental health outcomes among Indigenous young people; (2) the benefits of digital strategies to address SEWB; (3) Indigenous leadership’s responses to COVID 19 and (4) national government, non-government and Indigenous-led digital strategies.

### 3.1. Existing Inequities Impacting Indigenous Mental Health and Well-Being Outcomes

Several studies highlight the social and environmental inequities associated with existing health conditions, including food insecurity; poor access to water, sanitation and health services; and lack of adequate housing to support larger family groups, which increases COVID-19 risk [5,8,21]. Compounding these issues are the glaring inequities in affordable access to digital technologies necessary to enhance mental health and SEWB [35,36]. Access, ability and affordability issues all need to be addressed to help close the gap in access to services to improve well-being outcomes for Indigenous people across their lifespan [37]. One study reported that approximately three million Australians do not have access, capability, opportunity or cannot afford the internet, and only 63% of Indigenous people in Australia have access to internet at home compared with 91% of other Australians [38]. This has potential to adversely impact on the ability of young people to use digital technologies to access mental health services [35].

### 3.2. Mental Health and Well-Being among Indigenous Young People

The review confirmed there is growing concern regarding mental health impacts of the COVID-19 outbreak [39] including the potential for increased suicide deaths [40], particularly among vulnerable adolescents, prompting calls for a national youth mental health strategy [41] and a global youth response [42]. Social isolation is a major risk factor for illness, and severing of social connections in the current pandemic may evoke fears of facing the crisis alone [43,44]. Furthermore, young people have felt confronted in different ways. Vulnerable to information overload on social media platforms, many young people have felt responsible to protect families, and Elders, as “the knowledge holders of culture”; and to use these platforms to share information and maintain connections within families and communities (p. 17, [21]). Many young people have experienced increased stress and disruption to social and educational routines. Indigenous young people have the right to be supported and nurtured to promote their well-being and resilience. This support is needed now, to avoid and limit the deleterious effects of the pandemic, and to support recovery once the acute stage of the pandemic is over.

### 3.3. The Benefits of Digital Strategies to Overcome Health Inequities

It has been recognised for several years now that the internet is an important source of information, knowledge and access to health support, including mental health support for Indigenous young people. For instance, a Melbourne study found Indigenous young people were over represented in phone calls to Kids Helpline related to suicide and self-harm [45]. Another study found just over 31% of Indigenous young people who experienced psychological distress did not seek professional help, and compared to their non-Indigenous peer were three times as likely to report a lack of control over their lives; and nearly twice as likely to report a low level of self-esteem [46]. Indigenous young people in high distress were more likely to seek help from friends (63.6%), the internet (44.3%) and parent/s or guardian/s (43.5%), while a higher number turned to community organisations, social media or a telephone hotline for support [46].

The association between good mental health outcomes and access to digital technologies, including computers, the internet, smartphones and mobile applications (apps), has been identified in several national studies [47,48,49]. Online interventions such as cognitive behavioural therapy tools and apps to address mental illness, particularly depression and anxiety [23] and suicide ideation [50], have been shown to be acceptable and successful with Indigenous young people in Australia [47,51,52,53,54,55]. MindSpot national online services were found to be effective in the assessment and treatment of anxiety and depression among Indigenous people, providing a cost-effective means to overcome barriers to their mental health care [55]. A systematic review of research studies examining telehealth outcomes also identified improvements in SEWB and clinical outcomes, with Indigenous people reporting positive interactions and improved access to quality health services [56]. Service providers reported benefits of reduced travel and improved screening rates [56]. Other studies also confirm that, when e-mental health approaches are used to support or supplement existing health services, they can help to improve Indigenous people’s mental health and SEWB as well as advance their quality of care [57]. E-mental health can support practitioner training, culturally appropriate co-designed tools and resources, organisational leadership and support, and government investment [57]. Clearly, telehealth models of care have potential to improve access to health and mental health services and targeted screening programs for Indigenous people. Telehealth models of care are also likely to improve Indigenous outcomes and access to specialist services when facilitated in partnership with Aboriginal community controlled health services and public hospitals [56].

Indigenous young people have also reported increasing acceptability of e-mental health resources that have potential to improve mental health and well-being if they are well supported and integrated through existing services structures [23]. However, for online interventions to be effective, access to the internet and computer literacy are essential, as is the ability to use a range of digital platforms including mobile phones.

### 3.4. Indigenous Leadership Responses

Several articles reveal that 20 Aboriginal and two Torres Strait Islander leaders, including scholars and practitioners from Universities and Indigenous health, mental health, allied health and psychology peak bodies around Australia have worked with the Australian Government Department of Health throughout the pandemic to ensure that public health responses, communication and containment strategies are sensitive to the diverse cultural contexts of Indigenous Australians [21,58,59]. The Aboriginal and Torres Strait Islander Advisory Group on COVID-19 was convened in March 2020 to provide advice on culturally appropriate responses and recovery for COVID-19. It includes members from the Aboriginal Community Controlled Health Organisation (ACCHO) sector, State and Territory Government representatives and Aboriginal communicable disease experts [58] and is co-chaired by the National Aboriginal Community Controlled Health Organisation (NACCHO) with the Department of Health [59]. The taskforce has advised on primary-care-targeted actions, workforce needs, cultural safety, testing, evacuation and biosecurity measures, and community information provision. However, these initiatives do not specifically address the need to enhance digital technologies to support the mental health and well-being of Indigenous young people. The National Indigenous Australians Agency has also worked in close partnership with Indigenous communities and leaders and State and Territory Governments in developing and implementing an Indigenous response to COVID-19. This strategy ensured that Aboriginal health services had greater access to telehealth services, in line with the Australian government’s commitment to prioritise telehealth solutions in rural, remote and Indigenous communities [60,61]. However, there is no specific mention of strategies to link with Indigenous young people.

A national COVID-19 working party comprised of Indigenous leaders from around Australia was also convened by the Group of Eight Australian universities to provide advice to the government. The working party has produced an independent report for the government, addressing the specific mental health and SEWB needs of Indigenous people in Australia [21].This report outlines key recommendations including the need for governments to acknowledge and facilitate the right to self-determination; strengthen the health and mental health workforce; address social and cultural determinants of health and ensure equitable “digital and telehealth inclusion with immediate attention to an Indigenous helpline” (p. 3). It also recommends conducting evaluations that acknowledge data sovereignty.

The report also asserts the need for Indigenous governance to manage the coordination of COVID-19 recovery in communities “through equitable, needs-based funding to support strengths-based, place-based, Indigenous-led, and community-led initiatives that address the social and cultural determinants of health and well-being” (p. 1 [21]). While the Australian government has been swift to allocate resources to mainstream mental health and suicide-prevention services, there is a significant underspend on Indigenous services [62] to address the critical need for culturally responsive initiatives and online strategies to support young people [35]. The Australian government has pledged $461 million for a national strategy to prevent suicide and promote the mental well-being of young and Indigenous people. Of this, $395 million is allocated to Headspace, $51 million to existing mainstream parenting support programs and just $15 million to Indigenous suicide prevention [63]. As a consequence, Indigenous leaders are calling for adequate mainstream funds to be allocated to support the distinctive needs of Indigenous young people [64].

### 3.5. National Digital Strategies

The Australian Government allocated funding through the Better Access to Mental Health initiative, which commenced in November 2017 to provide e-mental health for all Australians living in rural and remote areas [63]. The Australian Digital Health Agency (ADHA) was established by the Australian national and state governments and territories to develop the digital health capability, innovation and integration in the health system across Australia. The vision is “for Better health for all Australians enabled by safe, seamless, secure digital health services and technologies that provide a range of innovative, easy to use tools for both patients and providers” [61]. After extensive consultation, the ADHA developed a National Digital Health Strategy (NDHS), which proposes seven strategic priority outcomes to be achieved by 2022. Based on the principle that all Australians have the right to digital health benefits, the strategic priorities are aimed at improving health system accessibility across the socioeconomic spectrum. In particular, acknowledging that digital health initiatives and services have the potential to empower patients and carers in rural and remote areas and vulnerable populations groups [65], addressing longstanding barriers to equity of access in healthcare.

The review identified four current initiatives that the ADHA is implementing in partnership with representatives from State and Territory Aboriginal and Torres Strait Islander health services and NACCHO. The ADHA meet quarterly to monitor strategic digital health priorities designed to contribute to the Australian Government’s Closing the Gap commitment [66]. On 4th December 2019, they met in Tasmania to develop strategies to improve digital health literacy and to ensure Aboriginal health services are partners in the work of the digital health agency. This partnership is essential to ensure that Indigenous holistic conceptions of health underpin the development, design and delivery of digital health technologies and services to improve communities’ access [67]. Under a national initiative led by the ADHA in partnership with Indigenous peak bodies, East Arnhem will become a community of Excellence for digital health [68]. The ADHA have committed $750,000 with a co-contribution of $240,000 from NT Health, Aboriginal Medical Services Alliance NT (AMSANT) and the Northern Territory Primary Health Network (NT PHN) to strengthen and embed digital health capabilities by promoting the My Health Record system, secure messaging, telehealth and medicines safety. Similar centres of excellence are also being implemented in remote towns in Western Australia and Queensland [69]. Given the impacts of COVID-19, the ADHA is regarded as the cornerstone of the health system to connect patients with health practitioners. However, it is recognised that many groups (including many Indigenous communities) still cannot access the benefits of digital connectivity. ADHA presented a road map of existing and emerging issues at a summit in Sydney in 2019 to finalise the detail around a high-level framework that encompasses three pillars for the future—digital literacy, new roles and ways of working in the health system and new models of care to transform the health system. By 2022, the NDHS expect to deliver the essential, foundational elements of health information that can be safely accessed, easily utilised and shared to support all Australians to make the right healthcare choices, have access to safe and personalised care and have support to benefit from innovative technologies [61,65]. However, currently there does not appear to be a specific focus on addressing the health and well-being needs of young Indigenous people.

In Western Australia, the trial of a new tool to help Indigenous people manage their healthcare via the GoShare digital platform has recently been launched [65]. However, as with the above strategies, there does not appear to be any specific focus on Indigenous young people. A review of resources across all states resulted in identifying a limited number of digital resources developed with and for Indigenous young people [70].

## 4. Discussion

Over the past months, social distancing measures and social isolation have severely limited opportunities for social engagement among young people. For many Indigenous young people, these connections are being maintained and sustained through the use of modern technologies to access mental health services and technologies (including apps), call lines and information as well as to maintain access to friend and families [23].

### 4.1. Digital Technologies, Connection to Culture, Community and Country to Enhance SEWB

Several studies have shown that the use of a range of digital technologies and social media contribute to strong cultural identity, enhance connections to community and country and improve mental health and SEWB outcomes [35]. The importance of Indigenous connection to culture, community and country underpinning Aboriginal SEWB is widely recognised within key Aboriginal health policy platforms in Australia [66,71]. Maintaining connections is even more critical in times of crisis [72]. Social connections are pivotal to the development of resilience in young Indigenous people [73].^.^ A South Australian study confirmed that positive family and peer relationships together with activities that facilitate “optimism and self-esteem” were associated with resilience and positive mental health outcomes [74]. Connection to family, community and country is being impacted by travel restrictions to and from discrete communities and social isolation expectations, which raises important questions: How can we adapt technology-based mental health services to include connection to culture and country as core elements to support connection for young people during the pandemic? How will we support young people to respond to loss and grief over the death of loved ones if restrictions to attend funerals and sorry business are re-imposed? How can young people be encouraged and supported to lead the way to respond to these issues through the innovative use of digital technologies?

The answers to some of these questions are already being explored by Indigenous academics, digital experts and technicians. For instance, the National Centre of Indigenous Excellence (NCIE) with Telstra Foundation have produced the first strategy for driving Indigenous digital excellence, based on a three-year consultation process with Indigenous digital makers, organisations and communities. The road map sets out the principles, goals and areas of focus and priority actions for an Indigenous Digital Excellence (IDX) future. During COVID-19, the NCIE IDX team successfully ran On Country Camps over five days with 30 participants from 11 communities via zoom. The team have developed culturally responsive resources operating across a range of platforms to keep young people linked together and connected with Elders, their families and communities. As participant, Elder Aunty Beryl said, “Modern technology is the way for us to keep going and keep connected” [29]. For example, young Indigenous people in Queensland are being encouraged to engage with their peers by uploading music and dance videos on YouTube [75].

Several studies and reports confirm the highly innovative ways in which young people share information and transformative ideas using digital technologies and social media to create and sustain virtual communities and networks [16,76]. They demonstrate how digital technologies and social media can contribute to strong cultural identity, enhancing connections to community and country [24,25,29]. Importantly, these studies show how access to digital technologies has the potential to facilitate healing, cultural continuity, self-determination and empowerment as well as address existing inequities in access. Similar strategies to support social connection and connection to culture, community and country, especially during times of extended trauma, anxiety, grief and loss, are urgently needed to promote SEWB and ensure resilience is maintained [77,78].

### 4.2. The Need to Support Digital Strategies to Address Inequities in Access to Health Services

Existing socioeconomic and health inequalities between Aboriginal and non-Aboriginal young people may be compounded by disparities in access to the internet. There is the potential for this disadvantage to be experienced by young people living in urban settings as well as in rural and remote settings. Census data reveal only 63% of Aboriginal young people aged under 25 years lived in a household with an internet connection compared with 91% of non-Aboriginal young people [38,79]. In 2019, digital inclusion for Indigenous peoples in both urban and rural locations was just 55% [80]. Young Indigenous people, including those affected by poverty, have limited access to the internet and requisite software and hardware. Many living in remote communities do not have reliable internet access due to the lack of necessary infrastructure within Indigenous communities. Being unable to leave their homes or communities to physically access health and social services, and barriers in access to social and emotional support via the internet, places Indigenous young people at greater risk of mental health threats such as depression, loneliness, hopelessness and anxiety. The closure of many schools, public libraries and internet cafes and the movement of schooling to online systems has been disruptive for young people living in families who do not have access to internet. This situation exacerbates the existing disadvantages and inequities being experienced by Indigenous young people.

The first wave of the pandemic revealed great strengths in terms of the Indigenous leadership, self-determination and resourcefulness of the Aboriginal Community Controlled Health Services (ACCHS) sector, as well as in the Australian health system and its commitment to support and be directed by the ACCHS in this time of crisis [81]. At the same time, it has laid bare the glaring inequities in housing, economic resources and basic community infrastructure that pose a risk to addressing health and well-being and ongoing social inequalities in Australia [82]. The pandemic has provided an opportunity for governments and service providers to take stock of the distinctive needs of Indigenous young people, their families and communities to be able to thrive, not just survive, in Australia now and into the future. Many remote Aboriginal communities and the ACCHS across Australia reacted swiftly and effectively to the COVID-19 outbreak. This illustrates both the heightened sensitivity of communities that expected to be disproportionately impacted by this crisis, as well as their resilience and self-determination. Indeed, globally, Indigenous communities are lighting the path to post-COVID recovery through strengthening connection to culture and community; highlighting the interconnection of people, place and planet [72]. It is essential both to understand the various ways in which Indigenous young people access and use social media and to monitor and evaluate its impacts and potential contributions in mental health interventions and improved access to mental health programs and services.

Our current research, Young Indigenous People’s Resilience and Well-being [83] focuses on the SEWB and resilience of young Aboriginal people in both urban and rural settings. Our early findings indicate that these young people experience high levels of psychosocial distress, racism and the ongoing impacts of social and economic determinants related to colonisation [83]. Further, our recent scoping review confirms overcoming such unrelenting adverse events and circumstances to be able to thrive, not just survive, is unique to Indigenous People in Australia [84].

## 5. Conclusions

Several studies in the review confirm that many Indigenous young people living in areas outside of urban sites are using the internet and social media. However, access to and use of the internet varies across groups and areas. Indigenous young people are no different. It is important that we develop a better understanding of these issues in order to support the development of policies that may assist young people to have better access to the internet and other digital platforms in times of crises.

There is a crucial need to develop and support digital initiatives to strengthen the mental health and SEWB of Indigenous young people in Australia. This requires the provision of adequate long-term funding to Aboriginal community controlled organisations in order to empower and learn from communities at this critical juncture in our history. Bringing together digital technologies with traditional customs and wisdom is testament to the innovation and leadership of Indigenous young people and the stewardship of Aboriginal Community Controlled Health Services.

We urge governments to adopt a trauma-informed, culturally responsive, integrated strength-based approach in the provision of mental health and well-being services including digital health solutions to support young people’s health, mental health and SEWB. For Indigenous young people, having a sense of social and cultural connectedness and a sense of control and mastery over their own lives is crucial to their well-being and resilience [85]. It is paramount for governments to work in partnership with Aboriginal peak bodies and leadership groups, to provide the financial resources and national online platforms necessary to implement strategies to improve access to health and well-being services, programs and activities to keep young people and their families connected to communities, culture, and country. While Australia is poised at this uncertain point in this pandemic, we all need to use this moment as an opportunity to “follow the lead” of Indigenous young people and turn a corner in addressing the inequities in the health and well-being of Indigenous communities. This requires enacting digital priorities outlined in Australia’s National Digital Health Strategy [61] in accordance with the United Nation Declaration on the Rights of Indigenous Peoples to self-determination [86]; the Charter of the United Nations and the International Covenant on Economic, Social and Cultural Rights [87]. It is critical that these rights and priorities are embedded in the National Youth Policy Framework currently being developed with input from Indigenous young people through the National Youth Taskforce [88].
